# Metabonomics Analysis of Myocardial Metabolic Dysfunction in Patients with Cardiac Natriuretic Peptide Resistance

**DOI:** 10.1155/2020/1416945

**Published:** 2020-12-09

**Authors:** Pan Chang, Shengping Lei, Xiaomeng Zhang, Jing Zhang, Xihui Wang, Juan Wu, Jianbang Wang, Jianping Geng, Baoying Chen, Jun Yu

**Affiliations:** ^1^Department of Cardiology, The Second Affiliated Hospital, Xi'an Medical University, Xi'an, China; ^2^Clinical Experimental Center, Xi'an International Medical Center Hospital, Xi'an 710100, China; ^3^Imaging Diagnosis and Treatment Center, Xi'an International Medical Center Hospital, Xi'an 710100, China

## Abstract

Brain natriuretic peptide (BNP) is an important biological marker and regulator of cardiac function. BNP resistance is characterized by high concentrations of less functionally effective BNP and common in heart failure (HF) patients. However, the roles and consequences of BNP resistance remain poorly understood. Investigate the effects of cardiac BNP resistance and identify potential metabolic biomarkers for screening and diagnosis. Thirty patients and thirty healthy subjects were enrolled in this study. Cardiac functions were evaluated by echocardiography. The plasma levels of cyclic guanosine monophosphate (cGMP) and BNP were measured by enzyme-linked immunosorbent assay (ELISA) and the cGMP/BNP ratio is calculated to determine cardiac natriuretic peptide resistance. Liquid chromatograph tandem mass spectrometry (LC-MS) based untargeted metabolomics analysis was applied to screen metabolic changes. The cGMP/BNP ratio was markedly lower in HF patients than controls. The cGMP/BNP ratio and ejection fraction (EF) were strongly correlated (*R*^*2*^ = 0.676, *P* < 0.05). Importantly, metabolic profiles were substantially different between HF patients and healthy controls. Kyoto Encyclopedia of Genes and Genomes (KEGG) analysis demonstrated that the differentially expressed metabolites are involved in signaling pathways that regulate cardiac functions. In HF patients, BNP resistance develops in association with a reduction in heart function and metabolic remodeling. It suggests possible functional roles of BNP resistance in the regulation of cardiac metabolism.

## 1. Introduction

Heart failure (HF) is a complex clinical syndrome characterized by structural disorders of ventricular filling or ejection and is unable to meet the blood supply needs of peripheral tissues [1, 2]. Importantly, it is estimated that approximately 38 million patients worldwide suffer from HF, being considered the main cause of morbidity and mortality, and its incidence is expected to continue to rise [3, 4]. The deterioration of cardiac function hampers the heart's ability to supply circulating blood, which in turn results in HF [5]. However, the specific pathogenic mechanism of HF and its complexity, known diseases such as myocardial infarction, hypertension, cardiomyopathy, heart disease, and oxidative stress caused by inflammation have been reported to be risk factors for the progression of heart failure [6, 7].

Brain natriuretic peptide (BNP) is a member of the natriuretic peptide (NP) family, which plays an important role, such as natriuresis, diuresis, vasodilation, and metabolic regulation [8–10]. All natriuretic peptides in humans contain a preserved ring structure consisting of 17 amino acid residues that form an intramolecular disulfide bond, and this structure is presumably responsible for receptor binding [11]. The receptors termed natriuretic peptide receptors (NPRs) are classified as type A or B. Binding of these guanylyl cyclase-coupled receptors leads to an increase in cyclic guanosine monophosphate (cGMP), which has downstream effects including diuresis and natriuresis, vasodilation, the inhibition of the renin-angiotensin-aldosterone system, enhanced myocardial relaxation, the inhibition of fibrosis and hypertrophy, the promotion of cell survival, and the inhibition of the inflammatory response [12–14]. Importantly, the resistance exhibited by natriuretic peptides participates in and acts as a molecular target for novel therapeutic approaches to combatting cardiometabolic disease [14, 15]. However, the underlying mechanisms are not well understood. Recent evidence indicates that natriuretic peptides possess functions beyond cardiovascular homeostasis that are related to the function and development of myocardial metabolites.

Metabolic dysregulation is one of the important components of the complex pathogenesis of HF [16–18]. Metabolic perturbation in HF is not just a disorder of the myocardium but also a systemic problem because changes in the composition of metabolites in circulation reflect the overall and systemic pathological changes, which is occurring in the failing heart along with changes in multiple organs and tissues throughout the body [19–22]. Metabolomics is a new systems biology approach that provides a platform for verifying metabolic molecules to distinguish patients with various stages of HF and analyze metabolic pathways at different stages of HF [23].

In this study, a metabolomics approach performed using liquid chromatograph tandem mass spectrometry (LC-MS/MS) was used to study the changes of metabolites in HF patients. The HF and healthy control groups were clearly distinguished by multivariate data analysis. Some metabolites were found to be significantly altered in HF patients, and the perturbed metabolic pathways were analyzed. Few studies have evaluated samples obtained from HF patients using metabolic analyses, which have shown that BNP resistance is correlated with metabolism in cardiovascular diseases. This study may, therefore, serve to identify possible metabolites that are differentially regulated between HF patients with natriuretic peptide resistance and may thereby provide a novel perspective in identifying possible metabolic biomarkers for disease screening and pathological studies. Thus, this study contributes to a better understanding of the changes that occur in cardiac energy metabolism during HF.

## 2. Materials and Methods

### 2.1. Chemical Materials

NH_4_OAc (ammonium acetate, LC-MS), NH_4_OH (ammonium hydroxide, LC-MS), and MeOH (LC-MS) were purchased from Sigma-Aldrich (St. Louis, MO, USA). Deionized water was produced by a Milli-Q system. The chemical standards were of analytical grade with a typical purity of 99%.

### 2.2. Study Subjects

The analyzed plasma samples were obtained from two cohorts (30 HF patients and 30 healthy controls (HC)). The 30 HF patients were diagnosed according to the criteria of the American College of Cardiology and the American Heart Failure (ACC/AHA HF) classification system. In particular, HF patients with hypothyroidism, systemic lupus erythematosus, inflammatory disease, chronic liver disease, chronic obstructive pulmonary disease, or cancer were excluded. Blood samples were collected before treatment. HC were included as HF-free subjects with no previous history of heart failure and no history of cardiac medication. All participants were recruited from Xi'an Medical University Second Hospital from January 2017 to January 2018. As showed in Table 1, the detailed clinical characteristics between the HF patients and HC are summarized.

### 2.3. Echocardiography

Echocardiography was performed noninvasively using a 55 MHz Vevo 770 transducer (VisualSonics, Canada) under 2.5% isoflurane gas anaesthesia. The measurements of wall thickness and left ventricular chamber diameter were obtained along the parasternal long axis in M-mode recordings of the left ventricle (LV). Then, LV fractional shortening (FS) and ejection fraction (EF) were calculated from the LV dimensions [24].

### 2.4. Blood Preparation

Approximately 5 mL of blood was drawn from each subject by venipuncture and stored in anticoagulant tubes of Ethylenediaminetetraacetic acid (EDTA) after the patients' admission from the hospital. After 30 min of coagulation at room temperature, plasma was separated by centrifugation (3000x g, 10 min). The 400 microliters of plasma were then placed into centrifuge tubes and stored at −80°C until required.

### 2.5. BNP and cGMP Test

Plasma was thawed prior to the quantification of cGMP and BNP levels, which were measured in the plasma of patients and HC using commercially available ELISA kits (R&D System, Minneapolis, USA and eBiosciences, Thermo Fischer Scientific, USA) according to the manufacturers' instructions.

### 2.6. LC-MS Analysis

The plasma was vortexed and thawed for 30 s. To measure metabolites, sample volumes of 200 *µ*L were extracted with MeOH : Acetonitrile (ACN) (1 : 1, v/v). The samples were then vortexed for 30 s and sonicated for 10 min. To precipitate proteins, the samples were incubated for 1 h at −20°C followed by 15 min of centrifugation at 20,000 g and 4°C. The resulting supernatant was removed and evaporated to dryness in a vacuum concentrator. The dry extracts were then reconstituted in 40 *µ*L/mg pro-ACN : H_2_O (1 : 1, v/v), vortexed for 30 s, and sonicated for 10 min. The extracts were centrifuged for 15 min at 20,000 rpm and 4°C to remove insoluble debris. The supernatants were then transferred to high-performance liquid chromatograph (HPLC) vials and stored at −80°C prior to LC/MS analysis.

Samples were separated on an amide column using mobile phase A consisting of water mixed with 25 mM ammonium acetate and 25 mM ammonium hydroxide and mobile phase B (ACN). The injection volume was 4 *µ*L, and the flow rate was 0.4 ml/min. Mass Spectrometry (MS) analysis was carried out on a Q-Exactive MS/MS device in both positive and negative ion modes. (1) The relevant tuning parameters used for the probe analyses were as follows: auxiliary gas heater temperature, 400°C; sheath gas, 40; auxiliary gas, 13; and spray voltage, 3.5 kV for positive mode and negative mode. The capillary temperature was set at 350°C, and the S-lens was set at 55°C. 2. The data dependent acquisition (DDA) method was as follows: full scan range, 60 to 900 (m/z); resolution for Mass spectrometry Imaging (MSI) and ddMS2, 70,000 and 17,500, respectively; maximum injection time for MSI and ddMS2, 100 ms and 45 ms, respectively; automatic gain control (AGC) for MSI and ddMS2, 3e6 and 2e5; isolation window: 1.6 m/z; and normalized collision energies (NCE): 10, 17, 25 or 30, 40, 50. 3. The full scan method was performed as follows: full scan range: 60 to 900 (m/z); resolution: 140,000; maximum injection time: 100 ms; automatic gain control (AGC): 3e6 ions [25].

### 2.7. Data Processing and Statistical Analysis

All MS raw data files (.wiff) were directly processed by the metabolomics processing software Progenesis QI (Waters Corporation, Milford, USA). Automated baseline filtering, peak recognition, integration, retention time correction, and peak alignment were carried out to generate a multivariable data matrix containing information on the retention time, mass-to-charge ratio, and peak areas. The data matrix was normalized by the sum of the areas of the identified peaks using the MetaboAnalyst 4.0 Web service. The data were subsequently subjected to multidimensional statistical analysis using SIMCA-P 14.0 software (Umetrics, Umea, Sweden). Principal component analysis (PCA) was first used to observe the overall feature distribution and dispersion among all groups. Orthogonal partial least-squares discriminant analysis (OPLS-DA) was performed to determine the distribution and dispersion among all groups. The metabolites with variable importance, indicated by a projection variable importance for projection (VIP) value greater than 1.0 and *p* values less than 0.05, were defined as differential metabolites [26].

The free databases KEGG and mzCloud were utilized to identify the metabolites obtained in the LC-MS analysis by matching them for accurate mass, fragment ion, and retention time. Finally, commercially available standards were employed to confirm the structures of the metabolites.

## 3. Results

### 3.1. Clinical Characteristics of Subjects

Initially, we consecutively recruited 30 HF patients and 30 healthy individuals as controls. The clinical characteristics of all subjects are shown in Table 1. There was no significant difference between HF patients and controls in age and body mass index. Plasma levels of low-density lipoprotein (LDL), cholesterol (CHOL), and triglycerides (TG) were higher, while high-density lipoprotein (HDL) was lower in HF patients than in controls [27].

### 3.2. BNP Resistance Occurred in HF Patients

**Figure 1:**
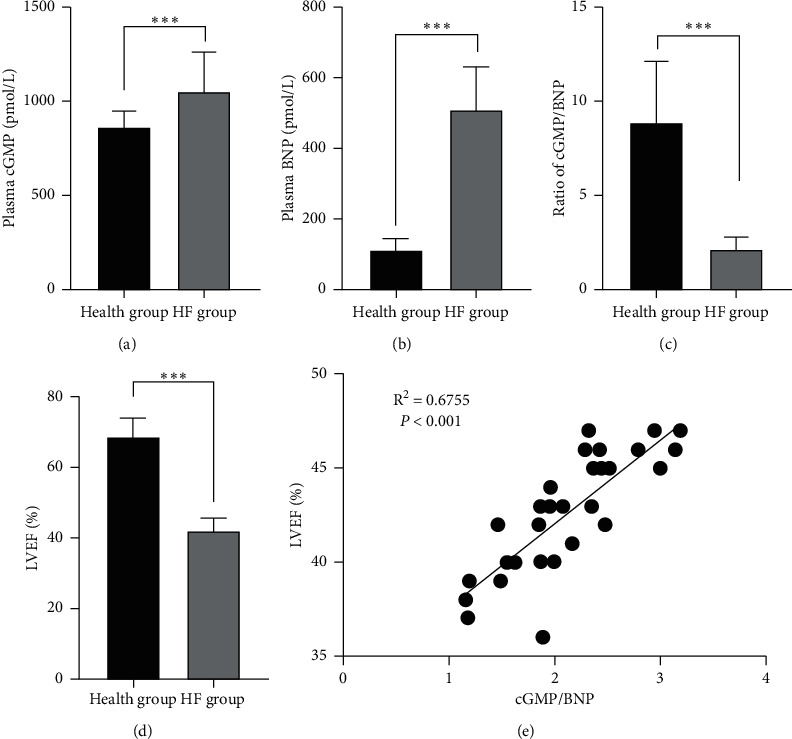
BNP resistance occurred in HF patients. (a) and (b) Plasma concentrations of cGMP and BNP. (c) The cGMP/BNP ratio. (d) Representative echocardiographic findings. (e) Representative correlation between LVEF and cGMP/BNP. BNP: brain natriuretic peptide; LVEF: left ventricular ejection fraction; cGMP: cyclic guanosine monophosphate; ^*∗∗∗*^*p* < 0.001 by Student's test.

As shown in Figures 1(a) and 1(b), plasma levels of cGMP and BNP in the HC group were significantly lower than those in the HF group (*p* < 0.001 for all healthy groups). Moreover, there was a good correlation between cGMP and BNP levels (Figure 1(c)), indicating that BNP resistance occurs in HF patients. Interestingly, there was also a good correlation between the cGMP/BNP ratio and EF, as shown in Figure 1(e).

### 3.3. Metabolic Profiles Are Aberrant in BNP Resistance

#### 3.3.1. Reliability of Metabolomics Data

**Figure 2:**
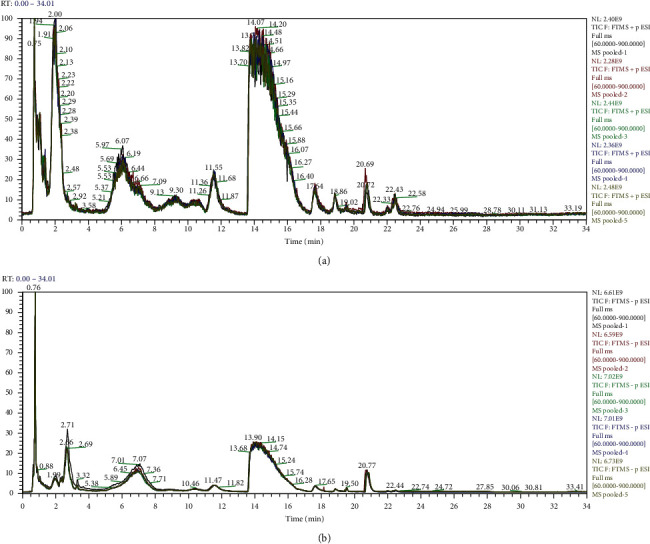
Liquid chromatograph-mass spectrometry results of experimental sample analyses. (a) Positive ion flow diagram for LC-MS detection of group samples. (b) Negative ion flow diagram for four LC-MS detection of group samples. LC-MS: Liquid chromatograph tandem mass spectrometry.

**Figure 3:**
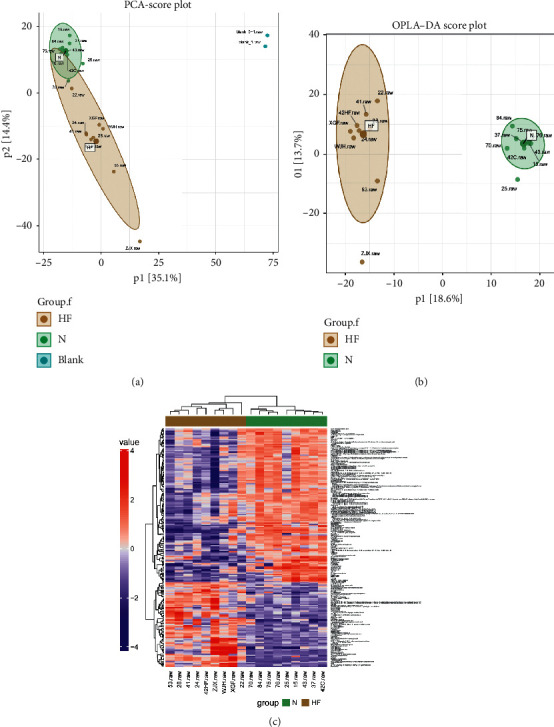
Plasma samples were obtained from HF patients and healthy controls to identify differential metabolomic characteristics by LC-MS. (a) Scatter plot of the PCA model for the HF group versus the healthy group. (b) Scatter plot of the OPLS-DA model for the HF group versus the healthy group. (c) Clustering heat map of the samples. The rows and columns represent metabolites and samples, respectively. LC-MS: Liquid chromatograph tandem mass spectrometry; HF: Heart failure; PCA: Principal component analysis; OPLS-DA: Orthogonal partial least-squares discriminant analysis.

In this study, quality control (QC) samples clustered together tightly in both groups, which indicated that the excellent QC repeatability of the two groups was good and the stability of the analysis system was high. Furthermore, the retention time change, the mass error, and the peak areas of QC samples showed that there was no difference between the two groups of samples, and the experimental study was feasible, as shown in Figure 2. Visualization of the features performed by PCA resulted in modes, as shown in Figure 3(a). The OPLS-DA score plots for HF patients in the HC group are shown in Figure 3(b) and it showed that this method had good discrimination and good instrument performance. The metabolic signatures of the HF and healthy groups were substantially different.

More than 1,000 molecular features were detected. 75 altered metabolites were significantly dysregulated between the HF and healthy groups, as shown in Figure 3(c). These data showed that metabolite clustering changed in parallel with HF status. Hierarchical clustering was formed based on the potential intervention targets. The rows show metabolites and the columns show samples. Colour keys indicate the expression value of the metabolite; green indicates the lowest expression and red indicates the highest. For each group, we calculated the Euclidean distance matrix of the quantitative value of the differential metabolites, clustered the differential metabolites by the complete linkage method, and displayed them on the thermodynamic diagram.

#### 3.3.2. Pathway Analysis

**Figure 4:**
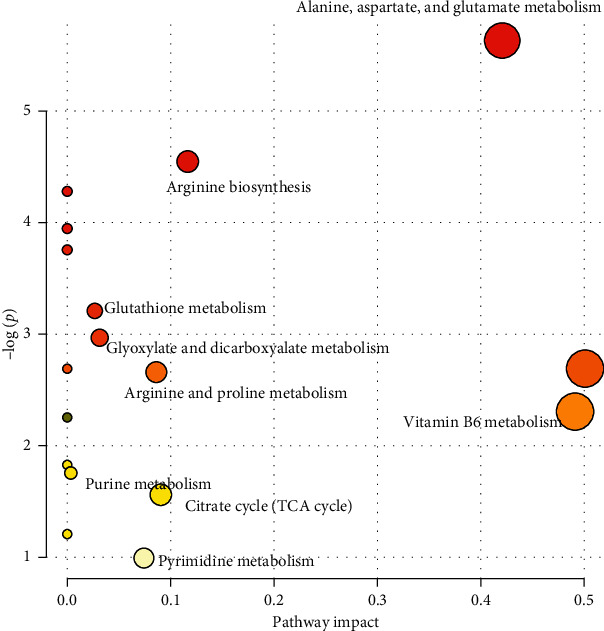
Metabolic pathway analysis showing the main pathways involved in heart failure. The plot shows pathways matched according to the *p* values in the pathway impact values from the pathway topology analysis.

In order to further explore the disease-specific metabolic relationships of the potential biomarkers, a more detailed pathway approach analysis was conducted. Pathway topology analyses were further conducted to explore the degree of metabolite folding changes and abundance in the list of important entities, and any metabolite is usually included in the list according to a fixed arbitrary threshold (such as the *p* value). The concentration tables are shown for the HF vs. healthy groups (Table 2). The results showed that 9 pathways that were significantly perturbed were related to HF; these included alanine, aspartate, and glutamate metabolism, glutathione metabolism, arginine, and proline metabolism, vitamin B6 metabolism, arginine biosynthesis, the citrate cycle (Tricarboxylic acid cycle, TCA cycle), purine metabolism, pyrimidine metabolism, and glyoxylate and dicarboxylate metabolism (Figure 4).

**Figure 5:**
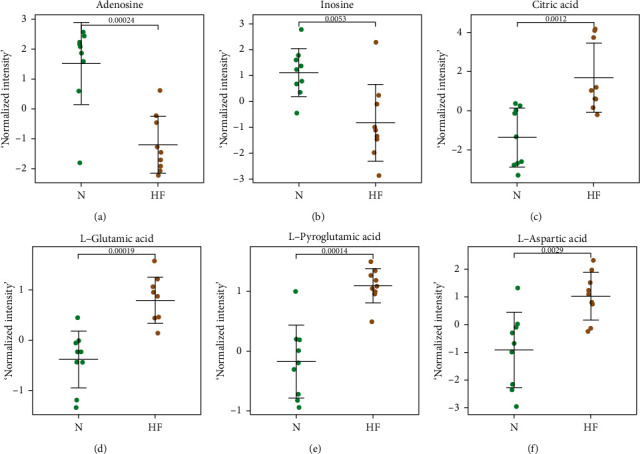
Scatter dot plots of potential biomarkers in the polar phase. All biomarkers shown here fulfilled the criteria of MV. MV criteria were a VIP value >1. Error bars indicate median and interquartile ranges. MV: Multivariate; VIP: Variable importance for projection.

Pathway analysis results indicated that the obvious disturbances observed in these pathways are related to HF, so they may play an important role in this disease and show their potential as HF biomarkers. We thus selected the following 8 identified metabolites for further study: citric acid, L-aspartic acid, L-pyroglutamic acid, pyridoxal, uracil, adenosine, citric acid, and L-glutamic acid (Figure 5), and we found that, except for adenosine, the content of all the other in HF patients was significantly lower than that of the control groups.

## 4. Discussion

HF is one of the most common and severe abnormal heart diseases, which leads to the loss of myocardial tissue and contractile force. Coronary artery disease determines a myocardial reduction in oxygen supply, which causes an impairment of myocardial contraction and relaxation [28]. Damage to the myocardial mitochondria that causes disordered mitochondrial metabolism during early reperfusion is a key mechanism underlying the overlapping occurrence of itself, as well as the other three in the progression of cardiomyocyte death, thereby leading to a number of acute myocardial ischemic patients with sustained substantial myocardial damage and even HF despite timely and successful reperfusion [29], but its early diagnosis or prognosis prediction still requires ideal biomarkers [30, 31]. The investigators have observed that patients with elevated cardiac filling pressure and clinical elevated HF have significantly higher BNP levels, which meant that HF is a state involving NP resistance. This concept is corroborated by the discovery that these patients also have reduced the cGMP/BNP ratios [32]. Interestingly, the levels of cGMP in plasma and urine are mainly related to NP-derived particulate guanylate cyclase-generated cGMP rather than nitric oxide-derived soluble guanylate cyclase-generated cGMP [14]. A previous study showed that NP resistance was observed in HF rats after the specific knockout of the NP receptor, and the correlation between cGMP/BNP and left ventricular ejection fraction (LVEF) was analyzed.

This study focused on the metabolomics study of two groups of HF patients with BNP resistance and serious impairment of cardiac function. The results showed that (a) impairment of cardiac function is correlated with BNP resistance and (b) different metabolic profiles are associated with cardiac function accompanied by BNP resistance.

In this study, a total of 75 metabolites were found to significantly contribute to the difference in plasma metabolism between HF with BNP resistance group and healthy groups. Not all of these differences could be assigned as chemical identities, but the use of database matching allowed the assumption that several metabolites are recognized. Amino acids are either ingested or endogenously synthesized in the human body and play important physiological roles as basic metabolites and metabolic regulators. Amino acids are key substrates required for mitochondrial metabolism and are usually elevated in HF groups [32, 33]. Glutamic acid, an amino acid, plays an important role in the creation of proteins. Glutamate is a very important excitatory neurotransmitter in the central nervous system; it is widely distributed; it is involved in individual cognitive, learning, and memory activities; it is closely related to the survival of nerve cells, synapse formation, and plasticity [34]. Glutamate is stored in glial cells in the form of glutamine, and the cyclic balance between glutamate and glutamine plays a crucial role in maintaining the normal function of brain cells. It would be interesting to explore the mechanisms that account for the increased glutamic acid in these patients. To generate energy for cardiac tissues, glutamic acid is converted into glutamate, which enters the glutamate-proline pathway [35, 36]. This amino acid is unique in that it is needed for nucleotide formation.

In this study, we found that the healthy and HF groups had common metabolic characteristics, as characterized by increased alanine, aspartate and glutamate metabolism, glutathione metabolism, arginine and proline metabolism, vitamin B6 metabolism, arginine biosynthesis, citrate cycle (TCA cycle), purine metabolism, pyrimidine metabolism, glyoxylate, and dicarboxylate metabolism, all of which are due to increased metabolic substrates and macromolecular precursors to meet the increased energy and protein synthesis needs of cell proliferation.

Our results suggest that the purine pathway could be associated with the progression of HF disease, and these metabolites may be developed into a biomarker analysis for HF diagnosis. Previous studies have reported that plasma uric acid is upregulated in patients with HF and is associated with HF burden. Another metabolite, adenosine, is involved in purine metabolism and has been reported to induce HF. Combined with the results of the current study, the purine metabolic pathway is strongly related to the progression of HF disease. Another important molecular group found in our results is critic acid, which is a weak acid that can be produced through the tricarboxylic acid cycle or introduced through diet. The evaluation of plasma citric acid is rarely used in the diagnosis of human diseases. In contrast, a urinary citrate excretion is a common tool for distinguishing kidney stones and renal tubular acidosis, and it plays an important role in bone diseases. The citric acid cycle, also known as the tricarboxylic acid cycle (TCA cycle) or Krebs cycle, is a series of enzyme-catalyzed chemical reactions that are vital to all aerobic organisms. The citric acid cycle also produces CO_2_ and several amino acids (aspartate, asparagine, glutamine, proline) and precursors of 1,4-Dihydroxy-2-naphthoyl-CoA (NADH), which are used in other important metabolic pathways.

We found seven amino acids upregulated in patients with HF, whose expression levels are highly correlated with each other. Three amino acids belong to the alanine, aspartate, and glutamate metabolic pathways, which occur mainly in the endoplasmic reticulum. Amino acids constitute the basic units of all proteins and serve as a substrate for energy-providing. At the same time, they have critical roles in cell signaling, biosynthesis, transportation, and key metabolic pathways. Interestingly, our results show that in untreated HF patients, L-glutamic acid and citrate acid were upregulated. This indicates that the levels of L-glutamic acid can be used as a potential indicator for the diagnosis and treatment effect evaluation of HF disease. In addition, amino acids and glucose are the main energy substrates for cardiomyocytes to produce Adenosine triphosphate (ATP), so the upregulation of L-glutamic acid in plasma may be related to amino acid intake or metabolic dysfunction in cardiomyocytes.

Taken together, the results of our study indicate that there is a unique metabolic characteristic that not only allows discrimination between HF patients and healthy groups but also allows BNP resistance with and without HF subjects, suggesting that the gradual depletion of energy reserves may be the cause of the development of cardiac impairment. Thus, our data indicate that energy metabolism is promoted in early/compensated HF states and that the depletion of metabolic capacity may lead to the progressive cardiac impairment observed in more serious cases of HF.

The early detection of HF with BNP resistance would provide an opportunity to implement appropriate therapeutic strategies to prevent or decrease unfavourable disease outcomes. In the present study, we employed untargeted omics analytical strategies to comprehensively analyze which molecules are dysregulated in the plasma of HF patients, and further ELISA assays verified the reliability of our omics data. Finally, HF-related metabolic pathway identification was used in biological information. Therefore, to the best of our knowledge, this study takes the lead in provide a reliable and comprehensive view of the plasma content profile of BNP resistance in untreated HF patients. Our results provide not only potential biomarkers for evaluating the diagnosis and prognosis of HF, but also clues for gaining a better understanding of the pathological mechanisms of HF.

## 5. Conclusions

In conclusion, we have demonstrated that metabolic disturbances occur in the plasma of HF patients with BNP resistance. We identified several dysregulated molecules, including metabolites. Our results could support the identification of novel biomarkers or effective therapeutic targets and provide clues as to upgrade the understanding of the pathology of HF. However, further validation of these candidate biomarkers in larger sample cohorts evaluated in double-blind tests is needed before they can be implemented in clinical diagnosis. In addition, the research function of biological experiments also needs to relate to illustrate the precise molecular mechanisms leading to the pathogenesis of HF.

## Figures and Tables

**Table 1:** Baseline characteristics of the study populations.

Variable	Health group (*n* = 30)	HF group (*n* = 30)	*p* value
Age (years)	69.47 ± 4.97	71.17 ± 6.41	0.2557
Male (%)	16(53%)	17(57%)	−
Body mass index (kg/m^2^)	23.64 ± 2.88	23.57 ± 2.58	0.9101
Biochemistry parameters			
** **LDL (mg/dL)	2.11 ± 0.19	2.90 ± 0.15	<0.01
** **HDL (mg/dL)	1.63 ± 0.14	1.46 ± 0.12	<0.01
** **CHOL (mg/dL)	4.19 ± 0.13	4.81 ± 0.17	<0.01
** **TG (mg/dL)	1.40 ± 0.09	1.65 ± 0.11	<0.01

Clinical characteristics of all subjects. Values are expressed as the mean ± standard deviation, median (first quartile, third quartile), or %. HF: heart failure; BMI: body mass index; LVEF: left ventricular ejection fraction; LDL: low-density lipoprotein; HDL: high-density lipoprotein; CHOL: cholesterol; TG: triglycerides.

**Table 2:** Identification of significant pathways in the HF and healthy groups.

NO.	Pathways	Total	Hits	*P* value	Metabolites	Impact
1	Alanine, aspartate and glutamate metabolism	28	3	0.0035	Citric acid, L-Aspartic acid, L-Glutamic acid	0.420
2	Glutathione metabolism	28	2	0.0402	L-Pyroglutamic acid, L-Glutamic acid	0.027
3	Arginine and proline metabolism	38	2	0.0700	4-Guanidinobutyric acid, L-Glutamic acid	0.086
4	Vitamin B6 metabolism	9	1	0.1000	Pyridoxal	0.490
5	Arginine biosynthesis	14	2	0.0139	L-Glutamic acid, L-Aspartic acid	0.1168
6	Citrate cycle (TCA cycle)	20	1	0.2095	Citric acid	0.090
7	Purine metabolism	65	2	0.1725	Adenosine, Inosine	0.0036
8	Pyrimidine metabolism	39	1	0.3695	Uracil	0.074
9	Glyoxylate and dicarboxylate metabolism	32	2	0.0514	Citric acid L-Glutamic acid	0.0318

## Data Availability

The data used in the study are provided upon request.

## Abbreviations

ACC: American college of cardiology
CAN: Acetonitrile
AGC: Automatic gain control
AHA: American heart failure
ATP: Adenosine triphosphate
BNP: Brain natriuretic peptide
cGMP: Cyclic guanosine monophosphate
CHOL: Cholesterol
DDA: Data dependent acquisition
EDTA: Ethylenediamine tetraacetic acid
EF: Ejection fraction
ELISA: Enzyme-linked immunosorbent assay
FS: Fractional shortening
HC: Healthy controls
HDL: High-density lipoprotein
HF: Heart failure
HPLC: High-performance liquid chromatograph
KEGG: Kyoto encyclopedia of genes and genomes
LC-MS: Liquid chromatograph tandem mass spectrometry
LDL: Low-density lipoprotein
LV: Left ventricle
LVEF: Left ventricular ejection fraction
MS: Mass spectrometry
MSI: Mass spectrometry imaging
NADH: 1,4-Dihydroxy-2-naphthoyl-CoA
NCE: Normalized collision energies
NP: Natriuretic peptide
NPRs: Natriuretic peptide receptors
OPLS-DA: Orthogonal partial least-squares discriminant analysis
PCA: Principal component analysis
TCA: Tricarboxylic acid cycle
TG: Triglycerides
VIP: Variable importance for projection.
